# “Reading the Mind in the Eyes” in Autistic Adults is Modulated by Valence and Difficulty: An InFoR Study

**DOI:** 10.1002/aur.2390

**Published:** 2020-09-15

**Authors:** Matias Baltazar, Marie‐Maude Geoffray, Christopher Chatham, Manuel Bouvard, Axelle Martinez Teruel, David Monnet, Isabelle Scheid, Eleonora Murzi, Sandrine Couffin‐Cadiergues, Daniel Umbricht, Lorraine Murtagh, Richard Delorme, Myriam Ly Le‐Moal, Marion Leboyer, Anouck Amestoy

**Affiliations:** ^1^ Centre d'Evaluation et Diagnostic de l'Autisme (CEDA) Centre Hospitalier Le Vinatier Bron France; ^2^ Roche Innovation Center F‐Hoffmann La Roche Ldt Basel Switzerland; ^3^ Université de Bordeaux Bordeaux France; ^4^ Pôle Universitaire de Psychiatrie de l'Enfant et de l'Adolescent Centre Hospitalier Charles‐Perrens Bordeaux France; ^5^ Centre National Pour La Recherche Scientifique INCIA UMR 5287 Bordeaux France; ^6^ Fondation FondaMental Créteil France; ^7^ UNIACT, NeuroSpin, CEA Université Paris‐Saclay Paris France; ^8^ Centre Hospitalier de Versailles CHV, Hôpital Mignot Pôle de Psychiatrie Adulte et Infanto‐Juvénile, Centre Expert TSAsdi Le Chesnay France; ^9^ INSERM, U955, Institut Mondor de Recherches Biomédicales, IMRB Laboratoire de Neuro‐Psychiatrie translationnelle Créteil France; ^10^ Institut Pasteur Paris France; ^11^ Psychiatry and Addictology Department, Université Paris Est Créteil, AP‐HP, DMU ADAPT Mondor University Hospital Créteil France; ^12^ Institut Roche Boulogne‐Billancourt France

**Keywords:** autism spectrum disorders, Reading the Mind in the Eyes, valence, difficulty, Generalized Linear Mixed Model

## Abstract

Autism spectrum disorders (ASD) are heterogeneous and complex neurodevelopmental conditions that urgently need reliable and sensitive measures to inform diagnosis properly. The Reading the Mind in the Eyes Task (or Eyes Test from now on) is widely used for this purpose. A recent study showed that subcategories of items of the children version of the Eyes Test could be especially discriminative to distinguish ASD and control children. Here, we analyzed the performance on the Eyes Test of 30 high functioning (IQ > 70) adults with ASD and 29 controls from the InFoR cohort multicentric study, using a Generalized Linear Mixed Model. We found that valence and difficulty modulate the performance on the Eyes Test, with easy and positive items being the most discriminative to distinguish ASD and controls. In particular, we suggest this result might be actionable to discriminate ASD patients from controls in subgroups where their overall scores show less difference with controls. We propose for future research the computation of two additional indexes when using the Eyes Test: the first focusing on the easy and positive items (applying a threshold of 70% of correct responses for these items, above which people are at very low risk of having ASD) and the second focusing on the performance gain from difficult to easy items (with a progression of less than 15% showing high risk of having ASD). Our findings open the possibility for a major change in how the Eyes Test is used to inform diagnosis in ASD.

**Lay Summary:**

The Eyes Test is used worldwide to inform autism spectrum disorders (ASD) diagnosis. We show here that ASD and neurotypical adults show the most difference in performance on *subgroups* of items: ASD adults do not improve as expected when comparing easy and difficult items, and they do not show an improvement for items displaying a positive feeling. We advise clinicians to focus on these comparisons to increase the property of the test to distinguish people with ASD from neurotypical adults.

## Introduction

Autism spectrum disorders (ASD) are neurodevelopmental disorders characterized by impaired social interactions and repetitive and restrictive behavior and interests [American Psychiatric Association, 2013]. Several theories have been formulated to explain the source of communication and social reciprocity alterations in autism, suggesting it could arise from poor cognitive or perceptual integration (i.e., having weak “central coherence” [Frith & Happé, 1994]), difficulties in making sense of social signals (i.e., being “socially blind”) [Baron‐Cohen, 2009]), or missing basic mechanisms that otherwise lead typical individuals to be spontaneously attracted to social signals (i.e., lacking “social motivation,” [Chevallier, Kohls, Troiani, Brodkin, & Schultz, 2012]. But despite these differences in primary causal mechanisms, they all hypothesize that deficits in perception or integration of nonverbal signals are present in ASD. Indeed, a strong body of research has established the presence of difficulties in processing others' nonverbal social cues and signals in autism, including facial expressions [Uljarevic & Hamilton, 2013] and gaze [Senju & Johnson, 2009].

These features of the syndrome led to the development of tools to assess the perception and understanding of social cues, in order to guide diagnosis and interventions. Among these, the Reading the Mind in the Eyes Task (or Eyes Test from now on) is of particular relevance, due to its ability to discriminate between typical adults and adults with autism without intellectual disability (i.e., high‐functioning autistic adults; [Baron‐Cohen, Wheelwright, Hill, Raste, & Plumb, 2001; Penuelas‐Calvo, Sareen, Sevilla‐Llewellyn‐Jones, & Fernandez‐Berrocal, 2019].

The Eyes Test contains 36 pictures of the eye area of male and female actors (in equal number). For each picture, participants have to pick the best complex mental state word out of the four displayed, while all words share the same emotional valence. The same team also designed a children version, where words were simplified to better suit the verbal level of young children [Baron‐Cohen, Wheelwright, Spong, Scahill, & Lawson, 2001].

Interestingly, some researchers have found that the ability to recognize facial expressions might be differentially impaired in autism, depending on the nature of the emotion and the valence that is displayed [Humphreys, Minshew, Leonard, & Behrmann, 2007]. For example, some publications point toward deficits in recognizing negative emotions such as fear or anger [Shanok, Jones, & Lucas, 2019]. The results of a recent meta‐analysis in this domain are less clear, showing an overall deficit in facial emotional recognition in individuals with ASD, independently of IQ or age, but with limited evidence supporting a modulation by emotional valence (except for a trend showing that fear might be less accurately recognized than happiness in ASD patients) [Uljarevic & Hamilton, 2013]. It is of note that this meta‐analysis was made on tasks presenting so‐called basic expressions [Ekman, 1993], reflecting the content of most studies examining emotion recognition in autism. The question we ask here is how could emotional valence affect the performance at the Eyes Test, a tool that includes very complex mental states such as “Pensiveness,” “Hostility” or “Playfulness”?

One study tested this very idea in children with autism [Baribeau et al., 2015]. Authors used the results from a previous independent publication [Koizumi & Takagishi, 2014] to parcel the items of the Eyes Test (Children version) in three valence categories (positive, neutral, and negative). They found that positive items were the most difficult to recognize out of all three categories for children with autism. They also found that positive stimuli discriminated better between patients and controls, while the overall difference across all items was not significant after controlling for IQ, sex, and age. These results suggest that the Eyes Test overall score might not be sensitive enough to differentiate individuals with ASD from controls, whereas the subscore for positive items might be. This is crucial information when considering the fact that clinicians have to use the most reliable and sensitive measures in order to proper inform diagnosis and treatment. Note that to the best of our knowledge, no psychometric linking study has been done to establish equivalence between children and adult versions of the test, so one should be cautious when applying conclusions from children data to adults and vice versa. But since both versions were designed with the same purpose in mind (i.e., measuring sociocognitive abilities based on verbal labeling of facial expressions), it seems reasonable to believe that results based on the children version are worthy to inform hypotheses to be tested on adults.

In the present study, we propose to apply a parcellation of items of the Eyes Test (adult version) by valence in accordance with previously published data [Harkness, Sabbagh, Jacobson, Chowdrey, & Chen, 2005] and to test individuals with ASD and controls comparable in terms of age, IQ, sex ratio, and educational level. We expected that participants with autism would perform worse than controls when considering all items. Based on children data, we expected that positive stimuli would induce higher differences between ASD patients and controls, compared to neutral and negative stimuli [Baribeau et al., 2015]. We also explored the influence of the difficulty of items [Baribeau et al., 2015; Lombardo et al., 2016].

## Methods

### *Participants*

A total of 59 participants (29 controls and 30 ASD participants) were included in the study (see Table 1). They were all part of the InFoR cohort. InFoR is a multicentric French longitudinal study, promoted by Fondation FondaMental, INSERM, and the Roche Institute. This cohort is constituted of a total of 117 individuals with ASD, including various age groups and levels of intellectual functioning, and 57 controls. Participants were followed during a 2‐year period and undertook various assessments at each yearly visit (clinical, neuropsychological, and biological) allowing for deep phenotyping of the cohort. Only adults from the cohort were included in the present study, using data from the initial visit only.

**Table 1:** Participant Characteristics

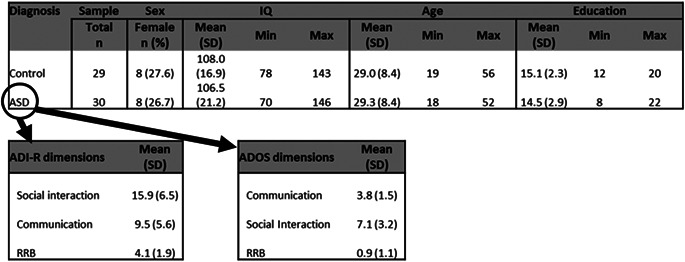

Abbreviations: ASD, autism spectrum disorder; Max, maximum; Min, minimum; RRB, restricted and repetitive behavior.

All adult participants were recruited by the Expert Center for Adults with ASD, which is part of the Adult Psychiatry Department of Mondor Hospital (AP‐HP, Creteil, France). All patients were adults seeking a diagnosis in a specialized center for the diagnosis of ASD. They all met diagnostic criteria for ASD [American Psychiatric Association, 2013]. ASD diagnosis was established by trained clinicians, based on a clinical interview and on the use of gold standard tools for assessing autistic features (i.e., the Autism Diagnostic Interview‐Revised (ADI‐R) [Lord, Rutter, & Le Couteur, 1994] and the Autism Diagnostic Observation Schedule (ADOS) [Lord et al., 2000]). Control participants were recruited through advertisement in the local press, at the local university and through Fundation FondaMental social network that promotes participation on research on mental health. Controls had no DSM‐IV Axis‐I psychiatric disorder and no first‐degree family history of schizophrenia, schizoaffective, autism or bipolar disorder (Diagnostic and Statistical Manual of Mental Disorders, 4th Edition, Text Revision; American Psychiatric Association, 2000). IQ was estimated using the full version of the fourth edition of the Wechsler Adult Intelligence Scale, at the exclusion of two patients who underwent the third edition (one with the full version and one with the abbreviated version). All participants were categorized as “high functioning,” meaning that their overall IQ was superior or equal to 70. Control and ASD participants were not different regarding age, sex ratio, IQ, or years of education (all *P* values superior to 0.37, see Table 1 for descriptive statistics), which is important given previous evidence of their impact on Eyes Test performance [Baker, Peterson, Pulos, & Kirkland, 2014; Kirkland, Peterson, Baker, Miller, & Pulos, 2013].

The study was part of the InFoR project, sponsored by INSERM (French National Institute for Health, Clinical Trial C07‐33), through a collaboration with the Roche Institute for Research and Translational Medicine. It was granted approval by local Ethics Committee or “Comité de Protection des Personnes” on November 14, 2008, authorized by the French authorities (ANSM B80738‐70 on August 11, 2008) and registered in a public trials registry (NCT02628808). All study participants gave their informed written consent to participation, in line with French ethical guidelines.

### *Experimental Design and Procedure*

#### *The Eyes Test*

A validated French version of the Eyes Test was used [Prevost et al., 2014]. The test contains 36 pictures depicting the eye region of actors displaying different facial expressions. Participants had to identify the mental state associated with the picture by selecting the most appropriate adjective out of the four displayed around the picture.

Items were subdivided into negative (*n* = 12), neutral (*n* = 16), and positive items (*n* = 8) following the work of Harkness et al. [2005].

Difficulty was determined by dichotomizing stimuli according to their associated percentages of correct answers in the norming sample (French adult version) [Prevost et al., 2014]. All stimuli with a percentage of correct recognition in Prevost et al.'s work superior to 70% (the median was 71%) were classified as Easy (*n* = 19) while the others were classified as Difficult (*n* = 17). This median split methodology is similar to previous dichotomizations of the Eyes Test items [Domes, Heinrichs, Michel, Berger, & Herpertz, 2007; Feeser et al., 2015; Radke & de Bruijn, 2015; Woolley et al., 2014].

At this point, we built a contingency table to check the repartition of items between Valence and Difficulty factors (see Table 2). This table shows a mostly even distribution when considering each factor alone. But it shows an unbalanced distribution of items when crossing the two factors. Indeed, the Difficult × Positive cell contains only one item, compared to four to eight items in other cells. That is why we focused our analyses on assessing the effect of each factor alone, without assessing their interaction.

**Table 2:** Distribution of Items per Valence and Difficulty Categories

Valence	Difficulty		Total (items)
	Easy (item no./total)	Hard (item no./total)	
Negative	5, 14, 22, 36/4 items	2, 11, 17, 23, 26, 27, 34, 35/8 items	12
Neutral	3, 9, 12, 15, 18, 24, 28, 32/8 items	4, 7, 8, 10, 13, 19, 29, 33/8 items	16
Positive	1, 6, 16, 20, 21, 25, 30/7 items	31/1 item	8
Total (items)	19	17	36

#### *Procedure*

The Eyes Test was proposed among several other evaluations in the context of the InFoR project (data not reported here, except for IQ, ADOS, and ADI‐R data). Stimuli from the Eyes Test were printed on paper and presented one by one. Participants had unlimited time to provide their answer.

### *Statistical Analysis*

All analyses were performed using the R software [R Core Team, 2019]. We computed a Generalized Linear Mixed Model using the *glmer* command of the lme4 package [Bates, Mächler, Bolker, & Walker, 2015] trying to predict performance at each Eyes Test item (1 = success and 0 = failure) by the following fixed factors: Valence (3 levels: Neutral, Negative, Positive), Difficulty (2 levels: Easy, Difficult) and Diagnostic Group (2 levels: Controls, Patients). We used a binomial link function and a maximum likelihood estimation method (Laplace Approximation). The interactions between Diagnostic Group and Valence and between Diagnostic Group and Difficulty were also tested. Due to the unbalanced distribution of items among the factorial design, we did not explore interactions between Valence and Difficulty, nor between Group, Valence and Difficulty. Participants were entered in the model as a random factor. The *α* value was set at 0.05 for all statistical tests. Significant effects were then explored by computing pairwise least square means contrasts, using the multcomp package [Hothorn, Bretz, & Westfall, 2008] and applying a Bonferroni correction for multiple comparisons to protect against Type I error. All reported *P* values are corrected for multiple comparisons throughout the text, tables and figures. Data were plotted using ggplot2 [Wickham, 2016].

A power analysis was conducted using the G*Power software (Version 3.1) [Faul, Erdfelder, Lang, & Buchner, 2007] and established that a total sample size of 50 participants would be needed to identify an effect of Group on overall Eyes Test performance. For this computation, we set the power parameter to 80% and derived the expected effect size from the Baron‐Cohen and colleagues' paper presenting the revised version of the Eyes Test [Baron‐Cohen, Wheelwright, Hill, et al., 2001].

## Results

**Figure 1:**
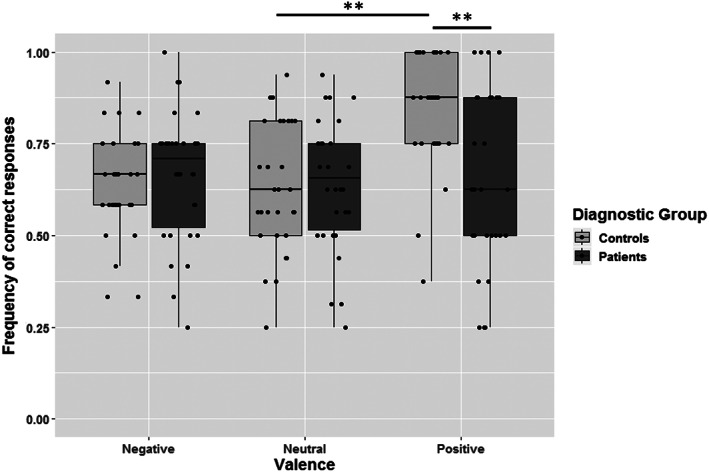
Eyes Test performance as a function of diagnostic group and valence of the items. *Note*. Individual data points are superimposed on boxplots. Performance was quantified as the frequency of correct responses, in order to obtain comparable scores for each valence category even though they contained different numbers of items. **P < 0.01.

**Figure 2:**
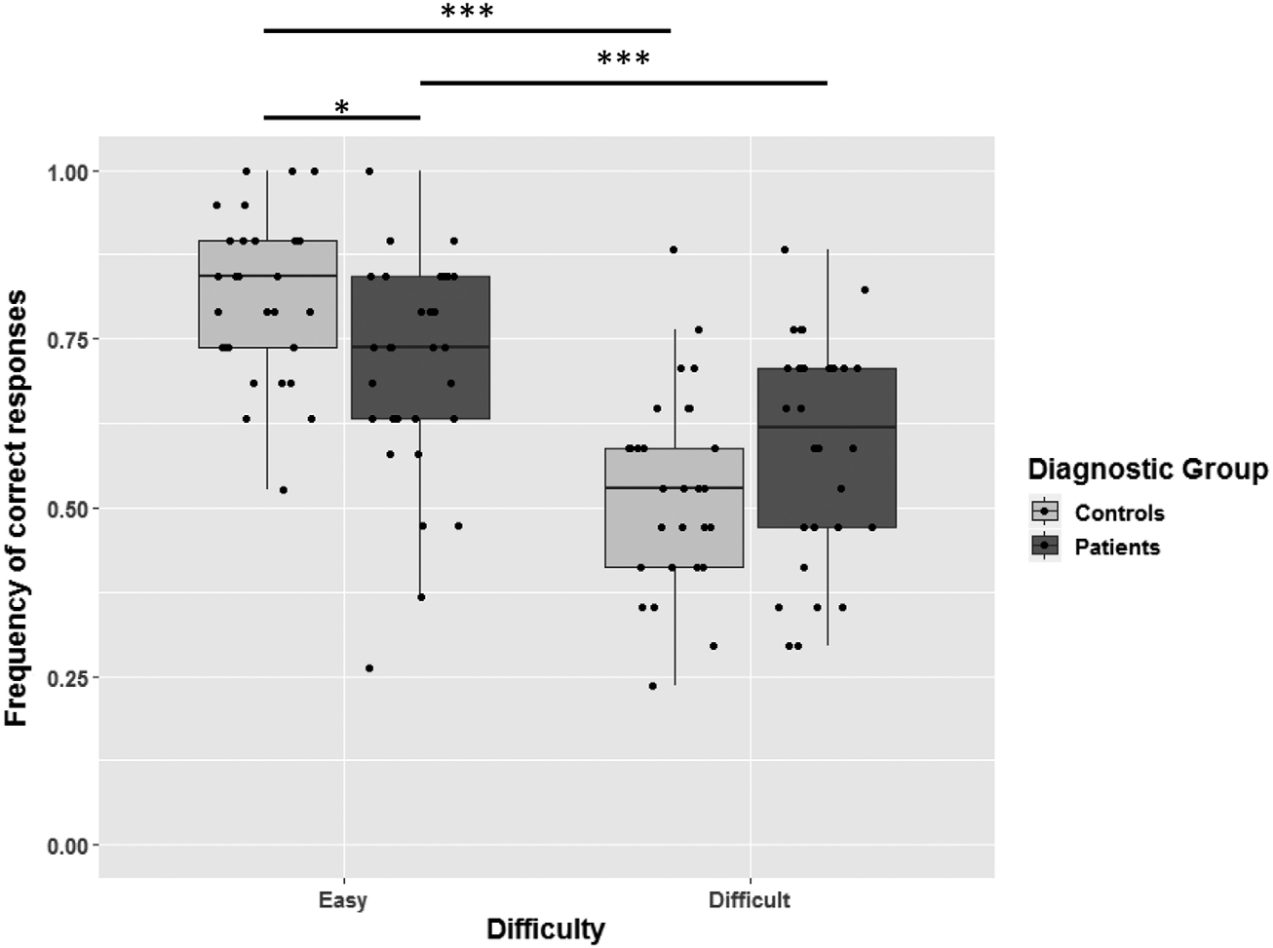
Eyes Test performance as a function of diagnostic group and difficulty of the items. *Note*. Individual data points are superimposed on boxplots. Performance was quantified as the frequency of correct responses, in order to obtain comparable scores for each difficulty category even though they contained different numbers of items. ***P < 0.001; *P < 0.05.

Descriptive statistics are displayed in Figures 1 and 2. We first assessed overdispersion in the Generalized Linear Mixed Model by using the overdispersion function by Bolker [2020]. Results indicated that data were not overdispersed (overdispersion ratio = 0.96; $\chi_{2115}^{2}$ = 2038.20; *P* = 0.88). Results of the GLMM showed that Eyes Test scores significantly differed as a function of Valence ($\chi_{2}^{2}$ = 6.01; *P* = 0.049) and Difficulty ($\chi_{1}^{2}$ = 82.28; *P* < 0.001). The effect of Group was not significant ($\chi_{1}^{2}$ = 0.02; *P* = 0.89). The interaction between Group and Valence ($\chi_{2}^{2}$ = 12.66; *P* = 0.002) and between Group and Difficulty ($\chi_{2}^{2}$ = 7.44; *P* = 0.006) was significant.

Regarding the Group × Valence interaction, multiple comparisons (number of comparisons = 15) revealed that Positive items led to significant differences between Groups (*Z* = 3.56; *P* = 0.008), while no significant differences were found for Negative (*Z* = 0.05; *P* = 1) and Neutral items (*Z* = −0.14; *P* = 1). Among Controls, only the difference between Positive and Neutral items was significant (*Z* = −3.67; *P* = 0.016). Among Patients, pairwise comparisons between Valence categories did not yield any significant results.

Multiple comparisons revealed that the Group × Difficulty interaction (number of comparisons = 6) was characterized by a significant difference between Groups for Easy items (*Z* = 3.19; *P* = 0.030), while the Group difference was not significant for Difficult items (*Z* = 0.12; *P* = 1). In concordance with the main effect of Difficulty, pairwise comparisons of Easy and Difficult items were both significant among Controls (*Z* = 8.11; *P* < 0.001) and Patients (*Z* = 4.64; *P* < 0.001). A careful examination of the *Z* values indicates that this difference was more pronounced for controls.

## Discussion

In the present study, we first replicated the effect of the valence of stimuli on the Eyes Test performance observed by Baribeau and colleagues in a sample of children [2015]. Specifically, positive stimuli led to better recognition scores in the controls group compared to neutral and negative ones, while no such difference was observed in individuals diagnosed with ASD. Secondly, we showed that easy items better discriminate between patients and controls, compared to difficult items, for which similar performance was found, a result that is also similar to what was observed in children by Baribeau et al. [2015].

It is of note that even though the effects were similar for adults (in the present study) and children (in Baribeau and colleagues' study, 2015), our effect was driven by control participants performing best for positive items, while the children effect was driven by individuals with ASD performing worse on easy items. Baribeau and colleagues proposed that due to the fact that younger children rely more on the mouth region to identify happiness on faces [Evers, Kerkhof, Steyaert, Noens, & Wagemans, 2014], children with a neurodevelopmental disorder such as ASD could show a relative immaturity in this aspect compared to age matched controls, which means that they rely more on the mouth region to identify positive facial expressions. This could explain their lower performance when identifying positively valenced items at the Eyes Test, since only the eye region is presented. Regarding adults, we propose that the rewarding nature of positively valenced faces, which is observed in neurotypical participants [Wang, Hahn, DeBruine, & Jones, 2016], could be associated with increased motivation, and thus higher resource allocation leading to higher performance. Evidence in the literature indicates that such a mechanism could be dysfunctional in ASD patients [Dichter, Richey, Rittenberg, Sabatino, & Bodfish, 2012]. Other evidence even suggest that displays of social affiliation, such as social touch [Peled‐Avron & Shamay‐Tsoory, 2017] or eye contact [Madipakkam, Rothkirch, Dziobek, & Sterzer, 2017; Trevisan, Roberts, Lin, & Birmingham, 2017], could be aversive for individuals with ASD. This could further explain why ASD participants did not show the improvement for positive stimuli in the present study, since such stimuli are associated with markers of sociality or cooperativeness (e.g., “Playful,” “Friendly,” or “Flirtatious”). Note that our effect could also be due to the fact that positive items were also easier, and that group differences were only observed for easy items in our adult sample (see below for a more thorough discussion on the matter).

In the present study, high‐functioning adults with autism seemed to be equally good as control participants at identifying complex mental states based on facial expressions from the eye region, while they seem to have difficulty in decoding easier and simpler stimuli. This could reflect the existence of two processes when decoding facial expressions: one intuitive, used for simpler expressions, for which ASD individuals could be particularly impaired; and one “cognitive,” based on conscious reasoning about the association of certain facial features and the state of mind they are supposed to reflect, for which high functioning ASD individuals could be equally good as controls. This interpretation is reminiscent of “dual systems” theories [Kahneman, 2011], and is further in line with data showing that high‐functioning ASD individuals display reduced intuitive reasoning and greater deductive reasoning in cognitive tasks [Brosnan, Ashwin, & Lewton, 2017; Brosnan, Lewton, & Ashwin, 2016]. Another explanation could be that due to an overall impairment in emotional facial expression decoding in ASD [Uljarevic & Hamilton, 2013], modulation of performance due to the difficulty of the items is less pronounced in this population, with maximum difference between patients and controls on easier items. We advocate in favor of this latter, more parsimonious hypothesis, but other studies are needed to better understand this effect (e.g., by asking participants to report their reasoning or lack thereof when solving items, by measuring reaction times to evaluate the cognitive cost of solving items, etc.).

The valence parcellation has been criticized in several studies, which found evidence in favor of a one dimensional structure of the Eyes Test [Carey & Cassels, 2013; see also Preti, Vellante, & Petretto, 2017; Vellante et al., 2013 for data on the Italian version of the test]. This brings higher validity to the hypothesis that the results obtained in the present study could be mainly attributable to the difficulty, and not the valence of the items. This also calls for nuanced interpretations in several studies investigating the effect of valence on Eyes Test performance [Fertuck et al., 2009; Frick et al., 2012; Harkness et al., 2005; Preissler, Dziobek, Ritter, Heekeren, & Roepke, 2010; Preti et al., 2017; Richman & Unoka, 2015; Savage & Lenzenweger, 2018; Schilling et al., 2012; Unoka, Fogd, Seres, Kéri, & Csukly, 2015; Wolkenstein, Schönenberg, Schirm, & Hautzinger, 2011]. For example, the meta‐analysis by Richman and Unoka [2015] indicates that patients with Major Depressive Disorders are impaired on the Eyes Test, with even more deficit for positive items, attributing this pattern to their being less able to decode rewarding positive facial expressions. But this could in fact reflect a more trivial process where easy items could be more informative in discriminating participants who are impaired (e.g., patients with major depression) from participants with no impairment in overall facial emotion recognition, while difficult items might lead to floor effects in both groups. To summarize this point, assessing the respective contribution of valence and difficulty independently in the adult Eyes Test might be problematic in some versions of the test.

It is of note that the Eyes Test overall score was not discriminative in distinguishing patients and controls in our study. This was not due to our study being insufficiently powered in this regard, as proved by the power analysis we conducted (see Methods section). This result is in contradiction with a recent meta‐analysis showing an overall significant difference between ASD patients and controls (including children and adult data [Penuelas‐Calvo et al., 2019]). A recent study by Lombardo and colleagues identified several subgroups based on their performance on the Eyes Test, using an unsupervised clustering approach on large samples of more than 600 patients and 200 controls dispatched in discovery and replication samples. Even though subgroups encompassing the majority of ASD patients did perform significantly worse compared to control participants, some subgroups of ASD and control participants did not differ between one another. As a tentative explanation for the null effect in our samples for the overall score, we propose that our ASD and control samples might be particularly enriched with individuals from these clusters showing weaker group differences.

Contrary to overall score, the easy items allow to distinguish patients and controls in our study, and the same result was found for positive items. This is crucial information since clinicians have to use the most reliable and sensitive measures in order to proper inform diagnosis and treatment. Instead of the Eyes Test overall score, we propose to use the performance gain from difficult to easy items, with participants progressing less than a threshold of 0.15 being at high risk of having ASD (Sensitivity = 70.0%; Specificity = 82.8%). Only considering the subgroup of easy and positive items could also be useful, with participants with frequency of correct responses of more than 0.70 on these items being at a very low risk of having ASD, due to the high specificity of this threshold (Sensitivity = 46.7%; Specificity = 93.1%).

Our study has several limitations. First, even though our sample size was enough to be able to detect overall Eyes Test score differences, it was still quite small regarding the interactions between diagnostic group and valence or difficulty. We used a restrictive Bonferroni correction to protect against Type I errors, but Type II errors might still have occur, thus requiring future replication studies with bigger sample sizes. This is especially true for the thresholds that we propose, which should be validated in independent and broader samples before being used in clinical settings. Also, our results might be generalizable to French populations only, with the French version of the Eyes Test. Second, we did not include a reaction time measure in our protocol. Such measure could be used to better discriminate between patients and controls. It could also be very informative about the underlying strategies deployed by patients and controls when performing the task, with shorter reaction times reflecting automatic strategies and longer reaction times reflecting more “cognitive” and controlled processes. Third, we did not include among controls a group with a psychiatric or neurodevelopmental condition other than ASD, so the assessment of the specificity of our measure is limited. For example, it is possible that patients with major depression could show the same pattern of results as our ASD sample (see [Richman & Unoka, 2015] for evidence in favor of strong recognition deficit of positive or easy items of the Eyes Test in patients with major depression and Baribeau et al. [2015] for similar evidence in patients with ADHD).

## Conclusions

In the present article, we show that the valence and difficulty of the items modulate the performance of adults with and without autism in the Eyes Test, with easy or positive items having the most discriminative power for sorting out individuals. In particular, we suggest this result might be actionable to discriminate ASD patients from controls in subgroups where their overall scores show less difference with controls. We propose for future research the computation of two additional indexes when using the Eyes Test: the first focusing on the easy and positive items (applying a threshold of 70% of correct responses, above which people are at very low risk of having ASD) and the second focusing on the performance gain from difficult to easy items (with a progression of less than 15% showing high risk of having ASD). These cutoffs have to be tested in broader independent samples to better assess their sensitivity and specificity. Our work opens the possibility for a major change in how the Eyes Test is used to inform diagnosis in ASD.
